# Case of Foreign Body in the Urethra

**Published:** 1916-11

**Authors:** G. Frank Lydston

**Affiliations:** Chicago, Ill.


### Case of Foreign Body in the Urethra.

BY G. FRANK LYDSTON, M. D., CHICAGO, ILL.

Man, twenty-eight years of age, clerk by occupation, was referred to me with the following history:

Ten hours previously, the patient inserted a wire nail, head first into his urethra. The nail escaped his fingers and disappeared into the urethra. Shortly afterwards, becoming alarmed, he sent for his family physician, who made rough and persistent but ineffectual attempts to extract it. The irritation of the foreign body and the traumatism incidental to the attempts at removal produced free urethral hemorrhage, followed by inflammation of the entire penis. There was complete retention of urine. As it seemed unwise to introduce instruments into the urethra under existing conditions, I made an effort to locate the nail by palpation. Careful examination of the urethra was negative until the index finger was introduced into the rectum, when the nail readily was felt, and could be clearly outlined. The nail lay straight in the canal, with its point free in the membranous urethra. The head of the nail apparently lay just within the sulcus at the vesical neck, where it plainly could be felt. The urethra was swollen and the prospects of extracting the nail were dubious. There also was the danger of pushing the nail into the bladder, thereby necessitating a cystotomy. The location of the nail was favorable to operation and it seemed unwise to delay it, since there was danger of the foreign body spontaneously becoming dislodged and entering the bladder.

Taking the foregoing points into consideration, I thought it best to immediately perform a perineal section, following the precedent of a precisely similar case in my practice.

I placed the patient upon my office table, and after asepticizing the perineum, I injected in the proposed line of incision 30 minims of a 5 per cent solution of novocain. A small quantity of 2 per cent cocaine also was injected into the urethra. A small incision was made in the perineal urethra upon the point of a medium-sized urethral sound. The incision was just large enough to admit the tip of the index finger. The opposite index finger was placed behind the head of the nail, from within the rectum, and the nail gradually worked forward through the membranous urethra until it could be felt by the finger in the perineal wound. The entire operation required about two minutes from the time the cutting began. Four interrupted catgut sutures were inserted in the urethral wound, the outer wound closed with horsehair, a small catheter inserted and the patient sent home in a taxi. Recovery was complete and rapid.

The nail removed was the ordinary wire nail, of the size known as ten penny. This is the second case of the kind that has come under my care and has been reported by me. Such cases are especially interesting, as showing the readiness with which the foreign body can be felt from the rectum, and the ease and simplicity with which, if necessary, a perineal section can be performed in the surgeon's office.

---

"How much does that stylish doctor of yours charge?"

"Ten dollars a visit."

"Gee! How often has he called at your house this month?"

"Twenty times."

"Gosh! You owe him $200 then?"

"Nope, only $10. He's made the other nineteen calls trying to collect it."—*Cleveland Leader.*

---

Here is one that was told by Congressman Benjamin G. Humphreys the other night in throwing the harpoon into a medical friend who was a fellow guest at a banquet.

Some time ago the keeper of a museum was engaged in placing some new curios that had just arrived from Egypt when he noticed a perplexed look on the face of his attendant.

"What's the matter, Smith?" he queried, going to the assistant. "Is there anything you don't understand?"

"Yes" answered Smith. "Here is a papyrus on which the characters are so badly traced that they are indecipherable. How shall I class it?"

"Let me see," returned the keeper, examining the curio. "Just call it a doctor's prescription in the time of the Pharaohs."—*Exchange.*

---

The teacher had explained to the whole school that the English sparrows had been brought to this country for the purpose of destroying the worms, particularly in our city parks. But now, she said, they have multiplied until they have become a nuisance themselves. "Children, which do you think is the greater nuisance now, the worms or the sparrows?" "I dunno," said Johnny Johnson, "I ain't never had the sparrows."—*Killeen Herald.*

---

The Wife—Oh, doctor, I think Henry is much better this morning. He took my hand just a minute ago and called me his own 'ittle tootsy wootsy.

The Doctor—The case is more serious than I thought. It's a very bad sign when a patient becomes delirious.—*New York World.*

